# Strengthening the evidence base on the use of digital health technologies to accelerate progress towards universal health coverage

**DOI:** 10.1093/oodh/oqae033

**Published:** 2024-09-09

**Authors:** Mathilde Forslund, Kirsten Mathieson, Yacine Djibo, Caroline Mbindyo, Neema Lugangira, Priya Balasubramaniam

**Affiliations:** Transform Health, c/o Impact Hub Basel Horburgstrasse 105, 4057 Basel, Switzerland; Transform Health, c/o Impact Hub Basel Horburgstrasse 105, 4057 Basel, Switzerland; Speak Up Africa, 18 Ave. Leopold Sedar Senghor, 8th Floor, BP 3837, Dakar, Senegal; Amref Health Innovations, Amref Health Africa, PO Box 30125, 00100, Wilson Airport, Nairobi, Kenya; Parliament of Tanzania, Bukoba, Kagera, Tanzania; Centre for Sustainable Health Innovations, Singapore Public Health Foundation of India, House No. 60, 4th Floor, Lane 2, 110030, New Delhi, India

**Keywords:** digital tools, digital health transformation, universal health coverage, health equity, primary healthcare, health outcomes

## Abstract

Digital health and the digital transformation of health systems are an enabler and accelerator to achieving Universal Health Coverage (UHC) in low- and middle-income countries. This can help close equity gaps, enhance primary healthcare, and improve efficiency and health outcomes. However, the digital health landscape is characterized by fragmented initiatives and investments, insufficient political will, and inadequate governance of health data. This special collection of *Oxford Open Digital Health* showcases impacts of digital health interventions and approaches towards expanded healthcare access and improved health outcomes. This commentary makes the case for policy innovation and leadership, improved investments, robust health data governance and a people-centred approach to leverage the benefits of digital health transformation. Prioritizing these areas can enhance the accessibility, affordability, and quality of care, towards UHC goals.

## INTRODUCTION

Despite progress, much of the world continues to lag behind in achieving the Sustainable Development Goals (SDGs), including target (3.8) to achieve Universal Health Coverage (UHC) by 2030 [1]. Progress towards the SDGs are marked with worrying trends with improvements in health service coverage stagnating over the last several years, alongside increasing levels of catastrophic out-of-pocket (OOP) health spending [2]. While low- and lower-middle-income countries have seen the greatest progress in terms of improved coverage, they nonetheless lag behind middle- and high-income countries and fare worse in terms of OOP spending. Inequalities in the coverage of health services remain, both between and within countries. Projections indicate that continuing with a ‘business-as-usual’ approach will delay achieving the SDGs until 2050 [3], including many health-related targets [4, 5]. The World Health Organization (WHO) has highlighted that without accelerated efforts, key targets for reducing maternal mortality and AIDS, tuberculosis and malaria will not be achieved by 2030 [6, 7].

Innovative and inclusive approaches and engagement models are increasingly needed to accelerate progress towards achieving UHC and wider SDG goals. An alternative to ‘business-as-usual’ is essential. The digital transformation of health systems offers just that: an opportunity to support countries in achieving their UHC and other health-related SDG targets.

Moving away from a focus mainly on the introduction of individual digital solutions and services, the ‘digital transformation of health’ refers to the multiple processes of integration of digital technologies and data into all areas that affect individual and collective health and well-being. This entails more than simply digitizing analogue workflows. It includes ensuring the necessary changes in the enabling environment, including legislation, regulation, funding, public awareness, understanding and involvement, to support the digital transformation [8].

This special collection of O*xford Open Digital Health* explores the impact of digital health interventions and approaches on facilitating key dimensions of UHC in low- and middle-income countries (LMICs). The research showcased seeks to answer such questions as: *What can we learn from evaluating digital health interventions and approaches in LMICs? How can these interventions expand health service coverage to marginalized communities and contribute toward UHC? What lessons can we draw from innovations in digital health implementation and governance?*

The research evidence and case studies in this special collection from The Philippines, Cameroon, Burkina Faso, Brazil and Kenya provide insights into how digital health interventions can impact health service provision at the local level. This includes broadening access and reach of health care for pregnant women in rural populations and providing telemedicine health services to marginalized communities. At the national level, we see examples of how digital interventions can enhance data-driven decision-making and ultimately improve health outcomes. Further research and case studies published through this special collection offer an important opportunity to continue to grow the evidence base on the impact of digital health on UHC.

## DIGITAL HEALTH AS AN ENABLER OF UNIVERSAL HEALTH COVERAGE

Digitalization and the use of digital tools have become a fundamental component of health systems and health service delivery. They serve as a key enabler and accelerator to achieving UHC in low- and middle-income countries. If implemented in an equitable, inclusive and sustainable way, the digital transformation of health systems has the potential to extend the coverage of health services and close equity gaps; strengthen primary healthcare; improve the efficiency and cost-effectiveness of health systems; and foster more inclusive health governance. The increased generation of health data resulting from digitalization can further enhance healthcare delivery and surveillance to improve health outcomes. For example, in this issue we see how the development of an International Patient Summary in Brazil can support continuity of care and informed decision-making.

### Extend coverage of health services and close equity gaps

Digital health solutions, such as telemedicine, point of care diagnostics and other digital health applications can help extend the reach of health services to underserved and marginalized populations, including youth, women, older people, and people living with disabilities [9]. Digital tools can help unlock barriers for populations in hard to reach geographies that may face obstacles in accessing health services. Digital health solutions can also help generate demand for specific health services, increasing health care uptake [10, 11]. Case studies in this special collection offer examples of digital health technologies expanding access to information and health care for key populations in Kenya and under-resourced communities in the Philippines [12].

### Strengthen health systems and primary health care

Digital transformation and tools can help improve the delivery, quality and efficiency of health systems and enable more effective system integration and continuity of care between service levels. A recent OECD report found that investments in digital transformation can fortify the foundations of health systems, strengthen resilience to public health shocks and contribute to stronger economies [13]. Countries with higher levels of digital adoption prior to the COVID-19 pandemic showed a higher positive trend in their handling of the pandemic and exhibited more decisive government action, and as a result potentially mitigated COVID cases and deaths [13]. A study in Cameroon highlights the advantages of using digital health platforms to enhance communication and care during pregnancy through remote consultations, increasing patient engagement and improving continuity of care and convenience [14]. While in Kenya, convergence around a national health information exchange (HIE) created momentum for the launch of a digitized primary care network in Kapkatet, Kericho County, Kenya [15].

### Improve efficiency and cost-effectiveness

Digital approaches can help streamline health systems, reduce redundancies, and improve budget management. Electronic health records (EHRs), for instance, enhance data accuracy and facilitate better care coordination, which can reduce healthcare costs. Modelling shows that scaling up digital solutions like EHRs in Nigeria, Kenya and South Africa could save 15% of health system costs [16]. If well-designed and implemented, digital tools can also help improve, streamline and support health financing functions such as revenue raising, pooling, purchasing, and benefits and entitlements [17].

### Foster more inclusive health governance

Digital platforms can increase opportunities for civil society and communities to participate in policy making, facility-level processes and governance. The active participation of women at all levels, including women’s leadership in digital health governance, ensures that their unique perspective and needs are considered, leading to more equitable health policies and outcomes. Additionally, creating platforms that facilitate women’s involvement in decision-making processes can enhance the inclusivity and effectiveness of health governance and helps to leverage the full potential of diverse leadership in driving innovation and accountability in health systems.

### Maximize health outcomes and public benefits of health data

Robust digital health systems enable better health surveillance, data collection and analysis. They can also help increase the availability and transparency of data, thereby enabling improved monitoring and accountability across the health system. There has been an exponential increase in the amount of health data being produced, used and stored due to digitization. If governed effectively, health data can improve public benefits and health outcomes through improved data-driven decision-making; advancements in research and innovation based on population-specific needs (e.g. tailored datasets on women); and improved health emergency and epidemic response.

## KEY CHALLENGES TO FULLY HARNESSING DIGITAL HEALTH TRANSFORMATION FOR UHC

The 202*1 Lancet and Financial Times Commission on governing health futures 203*0 found that the ‘digital ecosystem itself is an increasingly important determinant of health,’ [18] with both potential benefits and risks. Yet the Commission highlights the potential for cyberattacks and data breaches to compromise patient data and healthcare systems, algorithmic reinforcement of discrimination, and misinformation and disinformation, which can harm individuals and communities. The bottom line of the Commission is that weak governance of digital technologies is causing health inequities and compromising human rights [19].

Digital health interventions often assume that improving health is primarily a technical exercise or financing problem. The concept of ‘digital determinants of health’ recognizes that access to and use of digital health tools is itself influenced by social determinants. Lower socioeconomic status, lower digital literacy, and lack of access to technology can prevent marginalized populations, youth, women, older people and people living with disabilities from fully benefiting from digital health innovations, exacerbating health disparities [20, 21]. Additionally, biases in the data used to train artificial intelligence (AI) and machine learning models for healthcare can lead to algorithms that perform poorly or discriminate against underrepresented groups [22]. Digital tools must be designed with equity in mind and implemented thoughtfully to ensure they reduce rather than widen health inequities driven by social determinants.

In many LMICs the landscape is dominated by fragmented solutions, with only some examples of national-level scale and excellence along a single healthcare journey [23]. Many advances in digital health have been driven by individual solutions and pilots or responses to vertical health challenges, rather than looking at the overall needs of a health system. Investments have tended to be short-term, uncoordinated, and not driven by or aligned to any overarching or long-term national goal. This ultimately results in missed opportunities to fully leverage digital technologies to advance UHC.

Underpinning this fragmented digital health landscape are overarching challenges. To fully harness the opportunity of digital technologies to deliver health for all in the digital age, the priority must be on ensuring an equitable, inclusive and sustainable digital health transformation. Towards this goal, key barriers must be overcome.

### Low political will

Weak understanding and political support hinder the investment needed to strengthen the foundational areas and create enabling environments for digital health transformation. Current approaches are typically opportunistic, piecemeal, and fragmented, based on external incentives rather than being driven by national priorities and needs on the ground. This is also due to the absence in many countries of costed national digital health strategies to guide a country’s digital health transformation and provide a blueprint that external partners can rally behind.

### Unclear funding landscape and insufficient allocation of resources

The ‘pilot-itis’ widely recognized across the digital health sector is partly due to the incentive structure generated by both the legislative and regulatory environment, and by donor-driven funding flows. Short-term funding cycles and reliance on donor resources often undermine the long-term sustainability—the benefits and outcomes of digital innovations for health workers and patients [24]—of digital health initiatives.

Multiple actors invest in digital health solutions, platforms, and infrastructure, often without a shared standard of what constitutes ‘good’ investment. Without clear criteria to strive for and to assess projects, investments do not necessarily always align with national health priorities and systems-building in support of UHC.

There is also a lack of publicly available information on current funding levels for digital health, and an absence of systematic tracking of different funding streams, which in turn undermines the mobilization of additional resources, coordination of investment, and alignment around country priorities and needs. Without transparency across the funding landscape, it is difficult to identify where gaps exist or where there is duplication.

### Lack of inclusive and participatory approaches

Without intentional efforts to meaningfully engage communities and health workers in the design and implementation of digital tools, the many solutions that emerge will not meet the needs of users, and ultimately will not support in closing equity gaps in health access and outcomes. It is particularly important to address the needs of marginalized populations, youth, women, older people and people living with disabilities, who typically face barriers to accessing health services, and therefore risk being left further behind, resulting in even wider health disparities. Moreover, civil society is often not included in various levels of health system planning, execution and monitoring, let alone the development and implementation of digital health strategies.

### Weak legislative, regulatory and policy environment

Many countries lack robust legal and regulatory frameworks to guide the development and use of digital health technologies and health data, thus posing risks to data privacy and equity. While digital health technologies and data present enormous opportunities to improve health and well-being, they must be developed and used in ways that are aligned with public health values and prioritize equity and human rights. The rapid advancements in AI have created even greater urgency of the need to ensure strong legislation and regulation are in place.

### Inadequate digital public infrastructure and the digital divide

Weak digital public infrastructure, unreliable or inadequate power supply, network outages, as well as coverage gaps, affordability and digital literacy undermine the potential of digital tools to improve health access and outcomes [8]. Without addressing these foundational elements, digital health transformation may also exacerbate exclusion, widen the digital divide, and continue to leave some populations behind [10].

## RECOMMENDATIONS

To unleash the potential of digital health to accelerate UHC progress, coordinated, multi-sectoral action is needed.

### Prioritize digital health strategies that will deliver UHC

Governments and their partners should prioritize digital health as an integral part of UHC strategies, recognizing the transformative potential this offers to achieve health goals. This includes prioritizing greater investment in digitally enabled health systems alongside other key areas of investment for UHC. Governments should develop and implement costed digital health strategies, involving communities and partners in the process, and ensuring alignment with UHC goals. As we move away from siloed solutions to holistic national digital health systems, having clear and costed plans in place will ensure that funding is directed to the areas most needed to close equity gaps and deliver health impact.

### Increase and better-coordinate funding for digital health

*Closing the digital divide: More and better funding for the digital transformation of health’* (2022) [8] estimates that US$ 12.5 billion is needed over the next five years for the digital transformation of health systems in 78 LMICs. This is an ambitious but achievable target, representing only 1% of the annual government health spending of these countries—a small proportional investment relative to the wider health system. Even in the current constrained fiscal environment, countries should still be able to domestically finance 60–70% of this gap, with donors funding the rest.

What is needed is not simply more investment, but better coordinated and aligned funding, backed by better data and evidence of impact, as is set out in this journal issue. Better coordination also ensures scarce funding is not duplicative and is driven by the needs of end-users and underserved populations.

### Develop a standardized measure to track digital health investment and quantify the financial value of investment, leveraging existing tracking and reporting mechanisms

In addition to increasing investment, governments must prioritize action for better tracking, reporting and coordination of investment—a prerequisite to quantify and close the funding gap and to direct funding to the areas of highest-priority. This includes building consensus around ‘what’ kind of data should be tracked, as well as ‘how’ both domestic and donor investments in digital health transformation should be classified, for example within existing mechanisms such as National Health Accounts and the Organization for Economic Co-operation and Development’s Development Assistance Committee.

Integrating the tracking of digital health investment as part of routine processes for health systems planning and budgeting (e.g. the UHC Monitoring Report) can help to ensure that digital health investments reinforce the UHC agenda and provide a metric that advocates, civil society and health workers can use to hold governments to account so that funds reach the priorities and populations in need.

### Ensure strong regulation and legislation to create an enabling environment for digital transformation while protecting the rights and privacy of citizens

Governments should ensure legislation, regulations and policies are sufficiently robust to enable an effective and equitable digital health system that upholds citizen’s rights, safeguards data privacy and supports digital literacy.

In addition to ensuring the right protections are in place, robust health data governance is also needed to support responsible data use for public benefit and to build public trust in health data systems. The endorsement by governments of a global health data governance framework offers an important opportunity to establish a global standard and build consensus around what is needed. A recently developed, and widely consulted, model law on health data governance [25] provides an important step towards, and the foundation for, a global framework.

### Put people and communities at the centre of digital health

It is crucial to involve diverse communities in planning, implementing and monitoring of digital health strategies, policies and approaches. Groups that stand to gain the most from digitally enabled primary health care, such as children, youth, women, people with disabilities and marginalized populations, must be given adequate opportunities and resources to shape the digital health transformation and hold decision-makers accountable. This people and community-centred approach will ensure that digital health initiatives and tools are grounded in equity and human rights, respond to community needs, and ultimately support the goal of UHC.

### Build capacity for digital health implementation

There is an urgent need to build capacity for digital health implementation. This includes investing in the training and education of health workers, policymakers, and technology developers to ensure they possess the necessary skills and knowledge to effectively utilize digital health tools. There is also a need for initiatives that support the training and empowerment of women in digital health leadership roles. Furthermore, strengthening institutional frameworks for health data governance and promoting interdisciplinary collaboration are critical components of this capacity-building effort. Coordinated, multi-sectoral action is required to harmonize fragmented initiatives, increase political commitment, and ensure robust data governance. This approach will help create a sustainable digital health ecosystem that supports the equitable delivery of health services and advances the achievement of UHC.

### Invest in foundational areas to close the digital divide

To deliver health for all in the digital age and ensure everyone benefits from the digital transformation of health, investments and action in foundational areas and the building blocks of digital health are essential. This includes, for example, strengthening digital health infrastructure, enterprise architecture, and digital public infrastructure, as well as closing the digital divide, including digital access, affordability, literacy and skills.

## CONCLUSION

The lessons from this special issue illustrate that efforts to increase access to health through digital interventions can yield impact towards national-level digital vision and improve the patient interaction with the health system. Investing in the enablers of digital health transformation is also crucial: evidence from a previous supplement in this journal demonstrated that investing in a country’s digital health enabling environment can support effective pandemic response and contribute to the broader health system [26]. Ultimately, realizing this potential requires political commitment and policy innovation to ensure everyone can enjoy the benefits of digitally enabled care, as part of their right to health.

## Data Availability

No new data were generated or analysed in support of this research.
